# Supplementary material for: Wnt/β-Catenin Signalling and Its Cofactor BCL9L Have an Oncogenic Effect in Bladder Cancer Cells
Source: Int J Mol Sci. 2022 May 10;23(10):5319. doi: 10.3390/ijms23105319 (PMC9141496; doi:10.3390/ijms23105319)
Supplement: Supplementary file 1 [file ijms-23-05319-s001.zip › ijms-1678459suppl from proof.pdf]

Supplementary Data

Supplementary Table S1

Supplementary Table S2

Supplementary Figure S1

Supplementary Figure S2

Supplementary Figure S3

Supplementary Figure S4

Supplementary Figure S5

Supplementary Figure S6

Supplementary Figure S7

Supplementary Table S1. Primers used for gene expression analysis.

**Gene**	**Primer**	**Sequence 5'→3'**	**Annealing temperature**
AXIN2	fwd<br>rev	GCTGACGGATGATTCCATGT<br>ACTGCCCACACGATAAGGAG	**60°C**
BCL9L	fwd<br>rev	TTCAGAGGCCAAAGAGGTGG<br>CATGGCTGGGTCTGCTACAT	**60°C**
BIRC5	fwd<br>rev	TGAGAACGAGCCAGACTTGG<br>TGTTCCTCTATGGGGTCGTCA	**60°C**
LEF1	fwd<br>rev	TGCATCAGGTACAGGTCCAAG<br>ACGTTGGGAATGAGCTTCGT	**60°C**
MMP9	fwd<br>rev	GAGACGGGTATCCCTTCGAC<br>AGTTGGAACCACGACGCC	**60°C**
MMP14	fwd<br>rev	TCCAGCAACTTTATGGGGGT<br>TTCCCGTCACAGATGTTGGG	**60°C**
SP5	fwd<br>rev	TCAGCAGTTGCAGCGTGAA<br>AGTACAGCCACCCAGAAGAAG	**60°C**
Luc2	fwd<br>rev	CGCCATTCTACCCACTCGAA<br>TGTCCACCTCGATATGTGCG	**60°C**
NLucP	fwd<br>rev	GGGAGGTGTGTCCAGTTTGT<br>CTTCAGCCCATTTTCACCGC	**60°C**

Supplementary Table S2. Immunohistochemical H-score for BCL9L protein staining in bladder cancer samples. The expression of BCL9L was very heterogeneous in bladder cancer samples and for this reason, the H-score method was used for the assessment of the staining. The H-Score is calculated by the formula: 3 x percentage of strong staining + 2 x percentage of moderate staining + percentage of weak staining (Ishibashi et al. 2003).

**patient**	**non-dysplastic urothelium**	**dysplastic urothelium**	**NMIBC**	**MIBC**
**1**			0.01	1.7
**2**	0.8	1.0	0.9	0.5
**3**		0.6		1.0
**4**			0.01	0.9
**5**			1.0	1.7
**6**		0.9	1.2	1.4
**7**	0.3		1.1	0.9
**8**	1.2	1.6	0.15	1.1
**9**		1.4		1.6
**10**				0.5
**11**		1.0		1.0
**Mean H-Score**	**0.77**	**1.08**	**0.63**	**1.12**

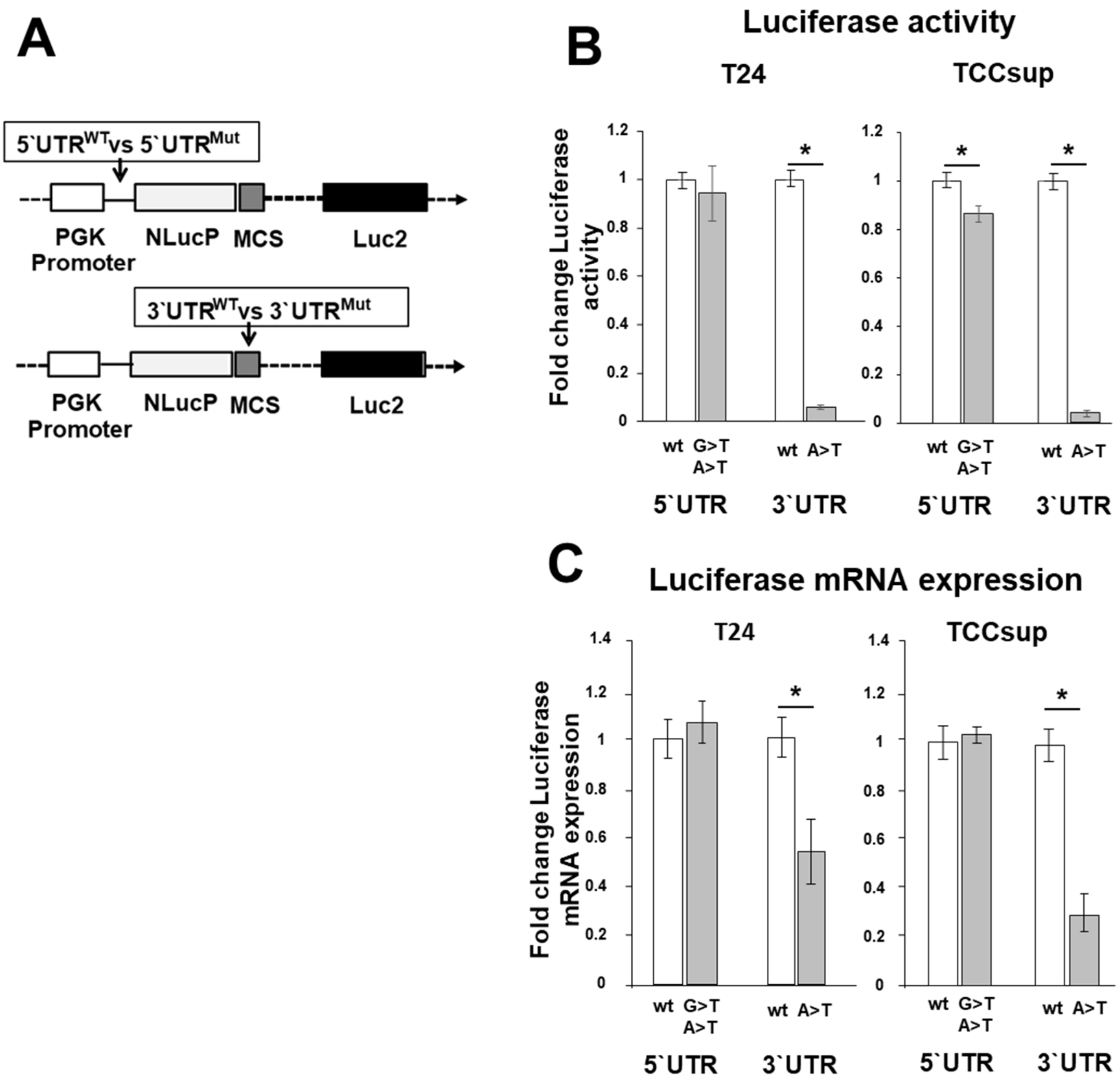

Supplementary Figure S1. Untranslated region (UTR)-mutation analysis of BCL9L by luciferase reporter assay. (A) Schematic view of the UTR sequence cloned in luciferase reporter. The wildtype and mutated 5′UTR sequences were cloned upstream, while the wildtype and mutated 3′UTR were cloned downstream of the NLucP reporter. The bladder cancer cell lines T24 (n = 4) and TCCsup (n = 4) were transfected with the luciferase plasmids and after 24 h luciferase activity (B) and mRNA (C) were analysed. The Luc2 luciferase was used for normalisation to eliminate variations from transfection. The data are expressed as mean ± standard deviation and statistical analysis was performed by non-parametric Mann–Whitney U-test with * p-value < 0.05. Values obtained from wild type sequences were set arbitrarily as 1. PGK: phos-phoglycerate kinase, MCS: Multiple Cloning Site.

Supplementary figure S2 below, continued on next pages, legend on the last page

Immunohistochemical staining of BCL9L in NMIBC and metachronous MIBC

NMIBC MIBC

patient 1

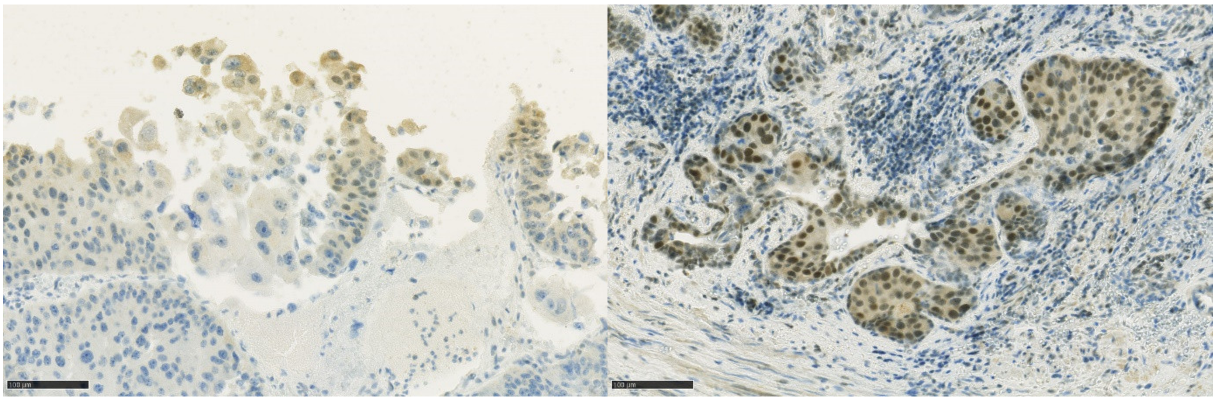

Few peripher tumor cells are weak stained (1% +, H-Score 0.01)

Homogenously weak to strong stained tumor areas (40% +, 50% ++, 10% +++, H-Score 1.7)

patient 2

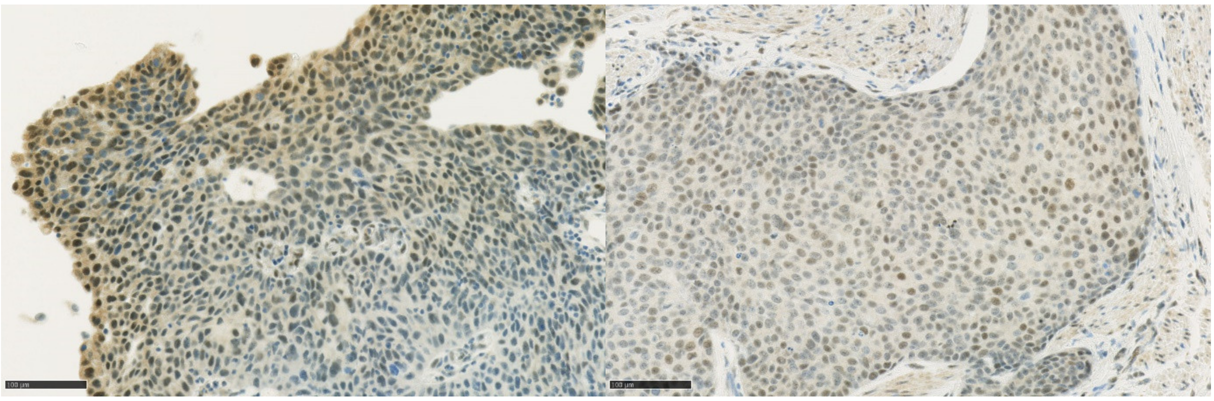

Peripheral increasing up to moderate staining (50% +, 20% ++, H-Score 0.9)

Homogenously weak stained tumor areas (50% +, H-Score 0.5)

patient 4

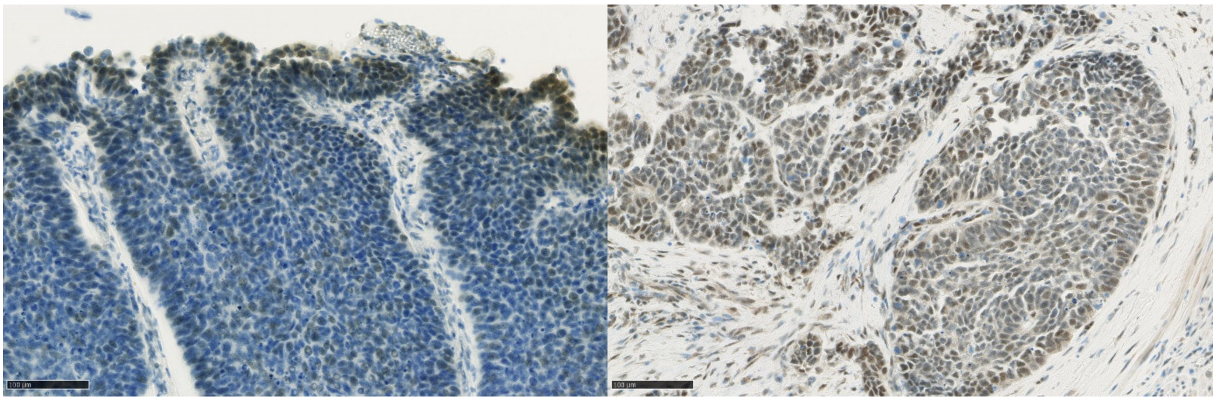

Few peripher tumor cells are weak stained (1% +, H-Score 0.01)

Homogenously negativ to moderate stained MIBC (50% +, 20% ++, H-Score 0.9)

patient 5

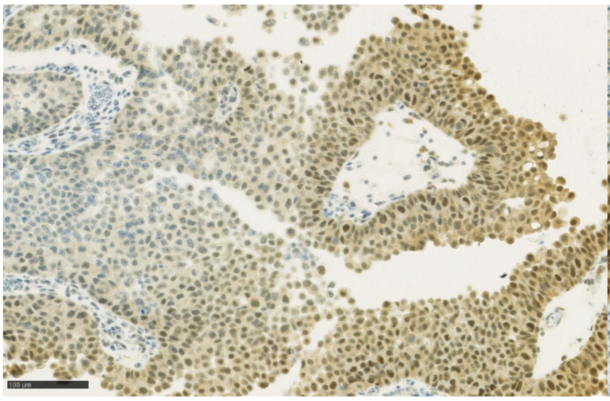

Peripheral increasing up to moderate staining
(40% +, 20% ++, H-Score 1.0)

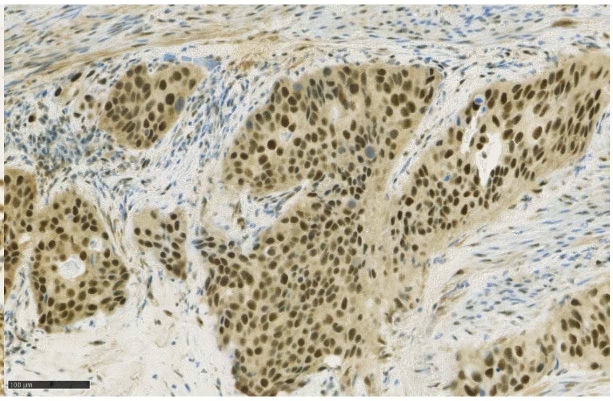

Mainly homogeneously stained tumor with few negative areas (10%, not shown). Positive areas are moderate to strong stained. (30% +, 40% ++, 20% +++, H-Score 1.7)

patient 6

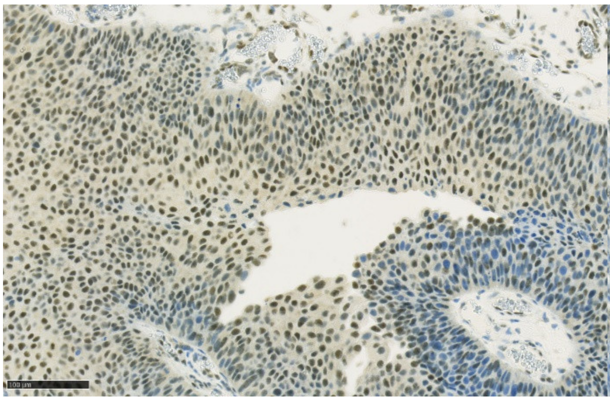

Heterogeneously stained tumor with negative, peripheral increased stained and completely positive tumor areas.
(40% +, 40% ++, H-Score 1.2)

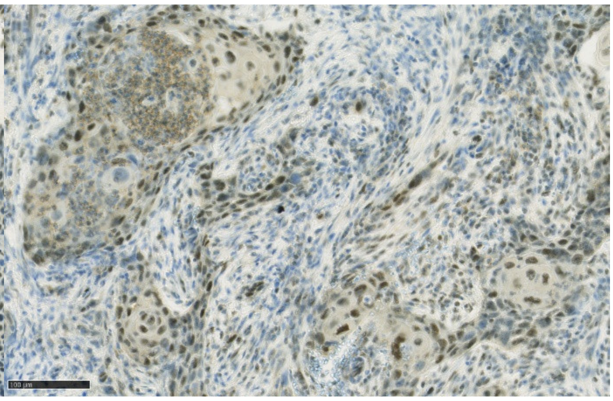

Homogeneously negative to strong stained squamous differentiated tumor.
(40% +, 20% ++, 20% +++, H-Score 1.4)

patient 7

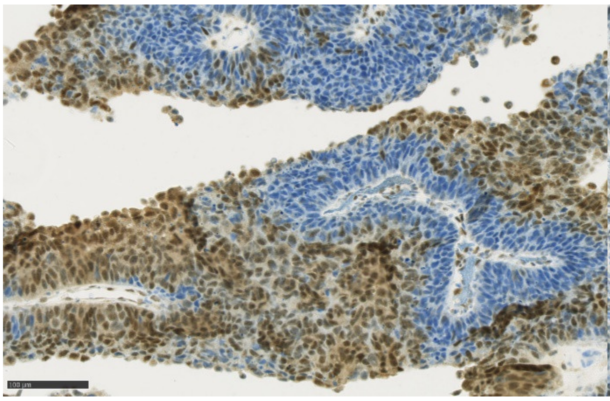

Heterogeneously stained tumor, peripheral increased stained and completely positive tumor areas.
(30% +, 40% ++, H-Score 1.1)

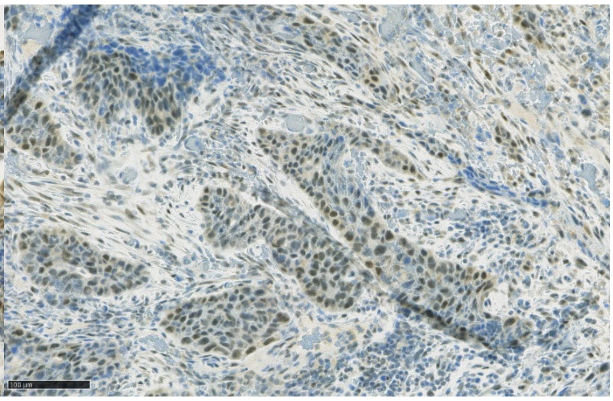

Homogenously negativ to moderate stained MIBC
(50% +, 20% ++, H-Score 0.9)

patient 8

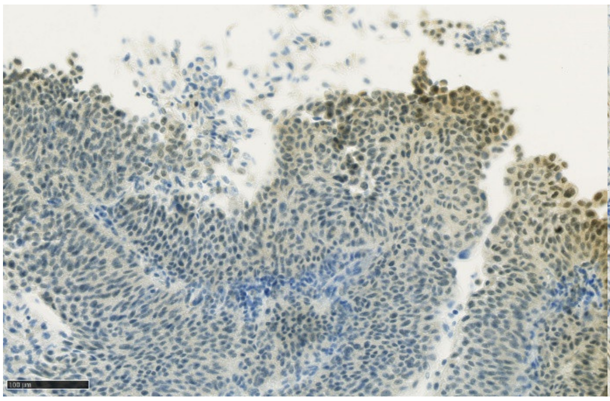

Only peripheral tumor cells are weak to moderate stained
(10% +, 5% ++, H-Score 0.15)

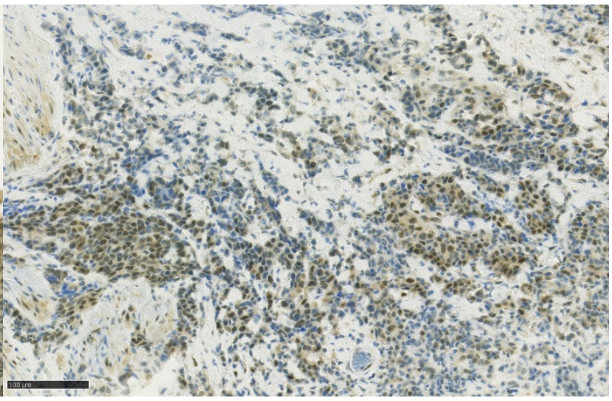

Heterogeneously stained tumor with some negative areas (20%, not shown). Positive areas are weak to moderate stained. (50% +, 30% ++, H-Score 1.1)

Immunohistochemical staining of BCL9L in further MIBC (NMIBC not available)

MIBC

patient 3

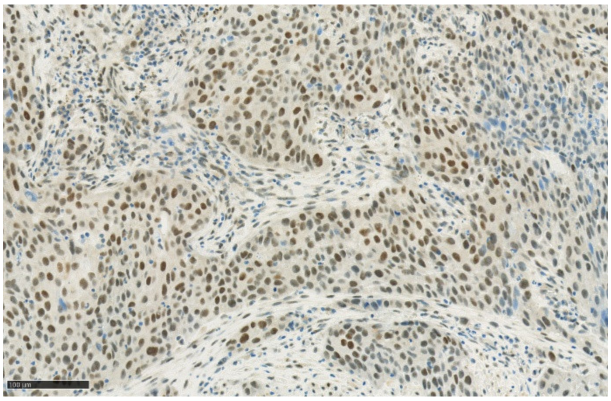

Heterogeneously stained tumor with some negative areas (30%, not shown). Positive areas are weak to moderate stained.
(40% +, 30% ++, H-Score 1.0)

patient 9

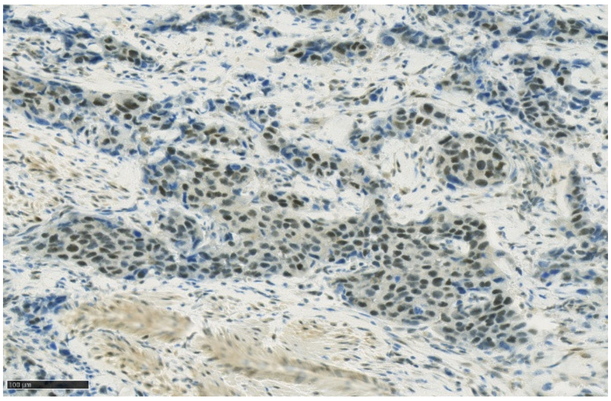

Homogeneously weak to strong stained.
(60% +, 20% ++, 20% +++, H-Score 1.6)

patient 10

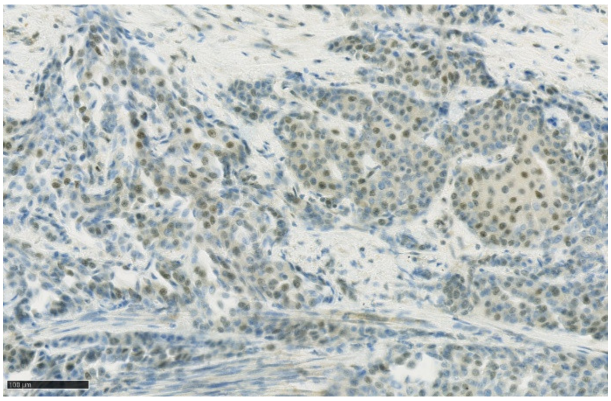

Homogeneously negative to weak stained.
(50% +, H-Score 0.5)

patient 11

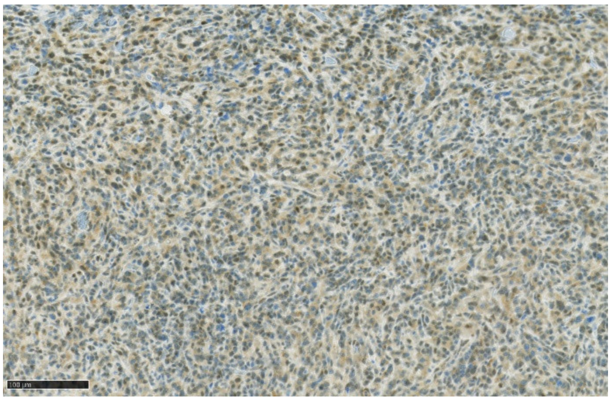

Homogeneously weak to moderate stained tumor cells.
(60% +, 20% ++, H-Score 1.0)

Immunohistochemical staining of BCL9L in non-dysplastic and dysplastic urothelium from tumor samples

normal-like (non dysplastic) urothelium

dysplastic urothelium

patient 2

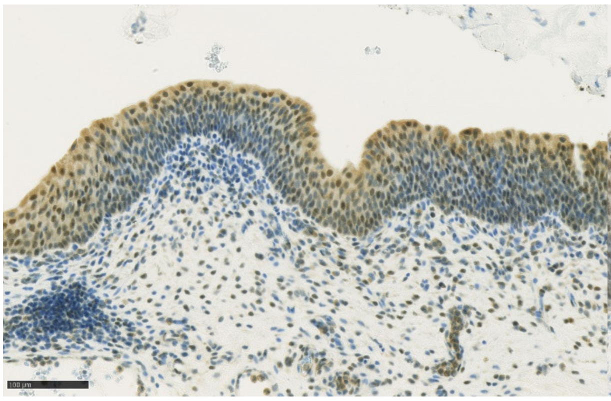

Weak to moderate stained normal urothelial cells.
(40% +, 20% ++, H-Score 0.8)

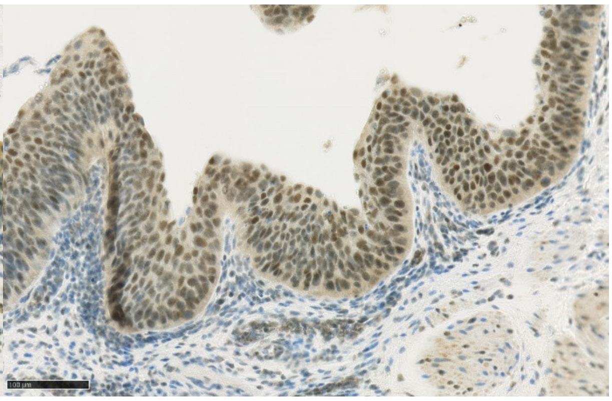

Weak to moderate stained dysplastic urothelium.
(80% +, 10% ++, H-Score 1.0)

patient 3

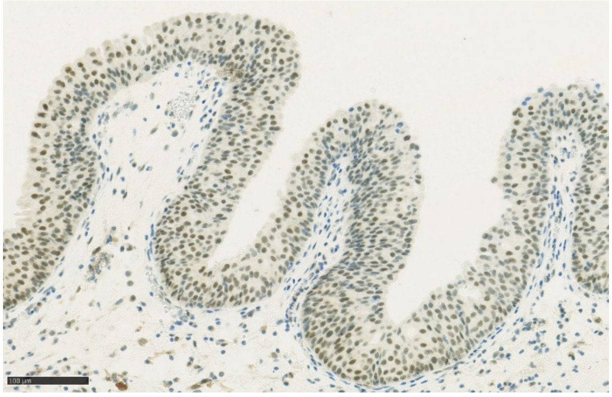

Negativ to weak stained dysplastic urothelium.
(60% +, H-Score 0.6)

patient 6

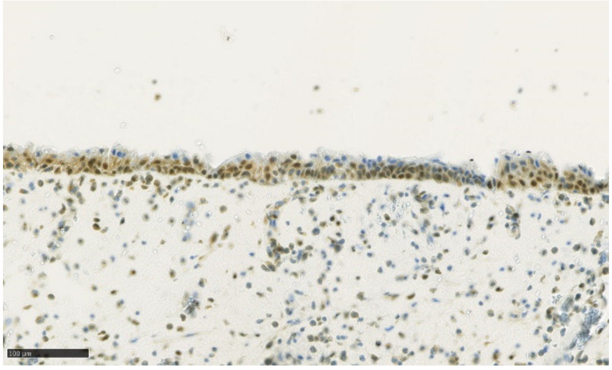

Moderate to strong staining of dysplastic urothelium.
(20% +, 20% ++, 10% +++, H-Score 0.9)

patient 7

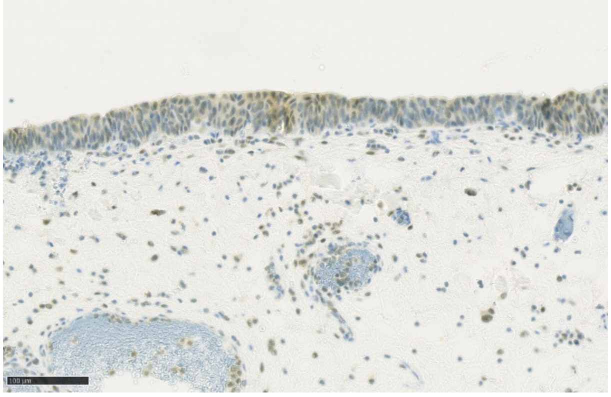

Negativ to weak stained normal urothelial cells.
(30% +, H-Score 0.3)

Patient 8

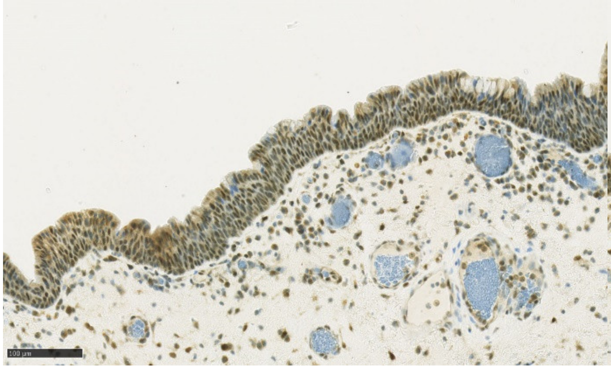

Weak to moderate stained normal urothelial cells.
(40% +, 40% ++, H-Score 1.2)

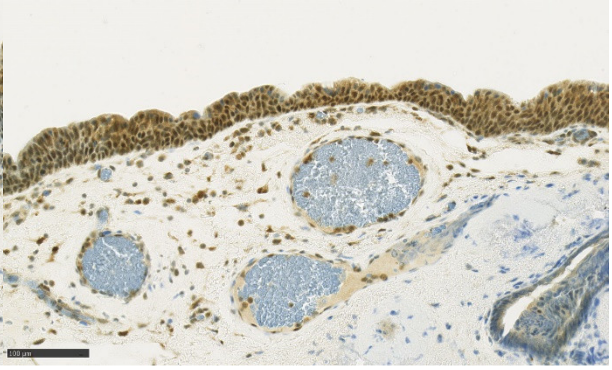

Moderate to strong staining of dysplastic urothelium.
(30% +, 50% ++, 10% +++, H-Score 1.6)

patient 9

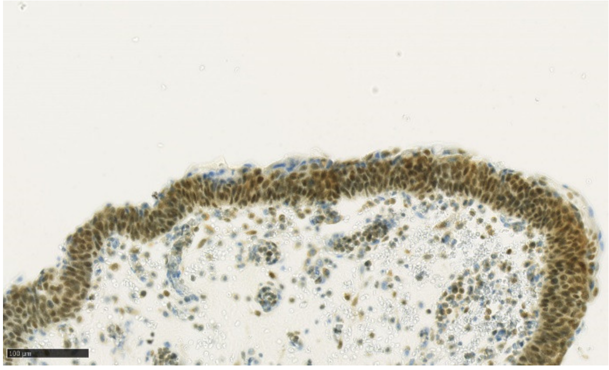

Moderate to strong staining of dysplastic urothelium.
(50% +, 30% ++, 10% +++, H-Score 1.4)

patient 11

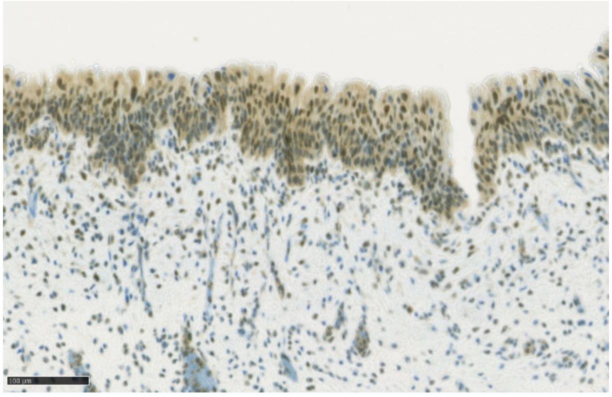

Weak to moderate stained dysplastic urothelium.
(50% +, 20% ++, 10% +, H-Score 1.0)

Supplementary Figure S2. Immunohistochemical staining of BCL9L in NMIBC, MIBC, dysplastic urothelium and non-dysplastic urothelium of patients with progressive Bladder Cancer. Scale bar = 100 µm. Overall average staining intensity of the tumor cells of the whole slide is specified as negative (-), weak (+), moderate (++) and strong (+++). The H-Score is calculated by the formula: 3 x percentage of strong staining + 2 x percentage of moderate staining + percentage of weak staining (Ishibashi et al. 2003).

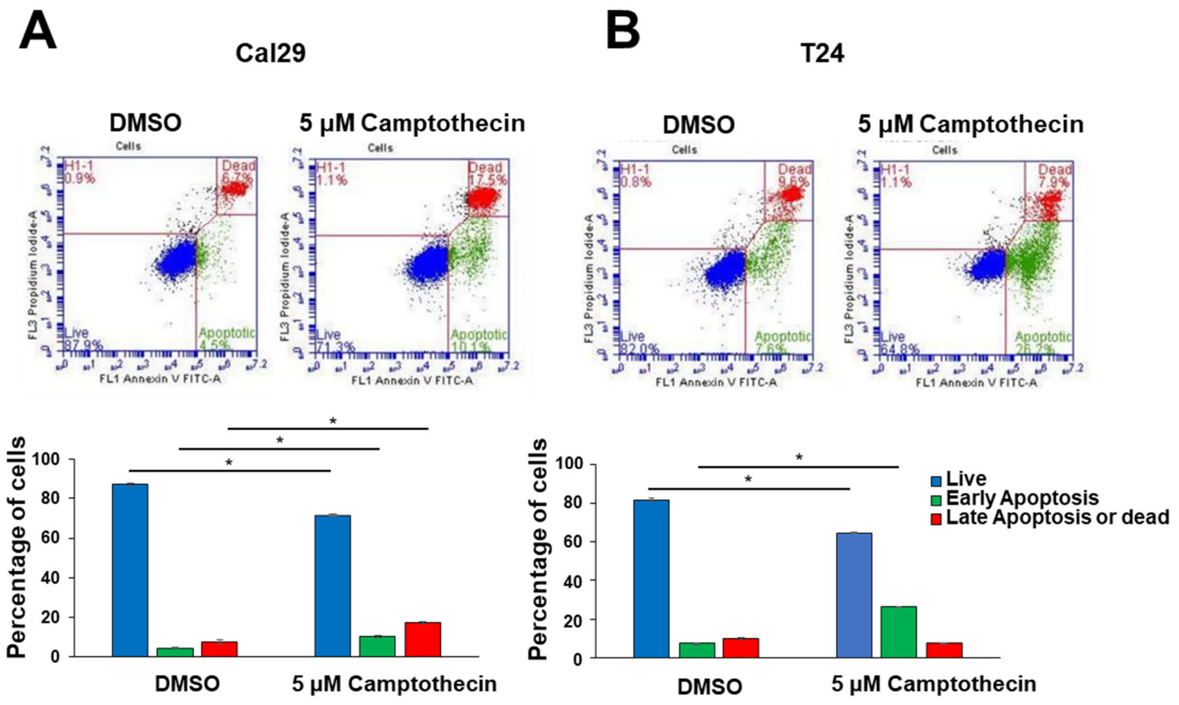

Supplementary Figure S3. Apoptosis analysis of bladder cancer cells after *BCL9L* knockdown. A) Cal29 and B) T24 cells were treated with 5 µM Camptothecin and were analysed for apoptosis after 24 hr. The apoptosis assay was performed by dual staining with annexin V-FITC and propidium iodide (PI) kit and analysed by flow cytometry. The data are expressed as mean ± standard deviation of three experiments and statistical analysis was performed by non-parametric Mann-Whitney U-test with * p-value < 0.05.

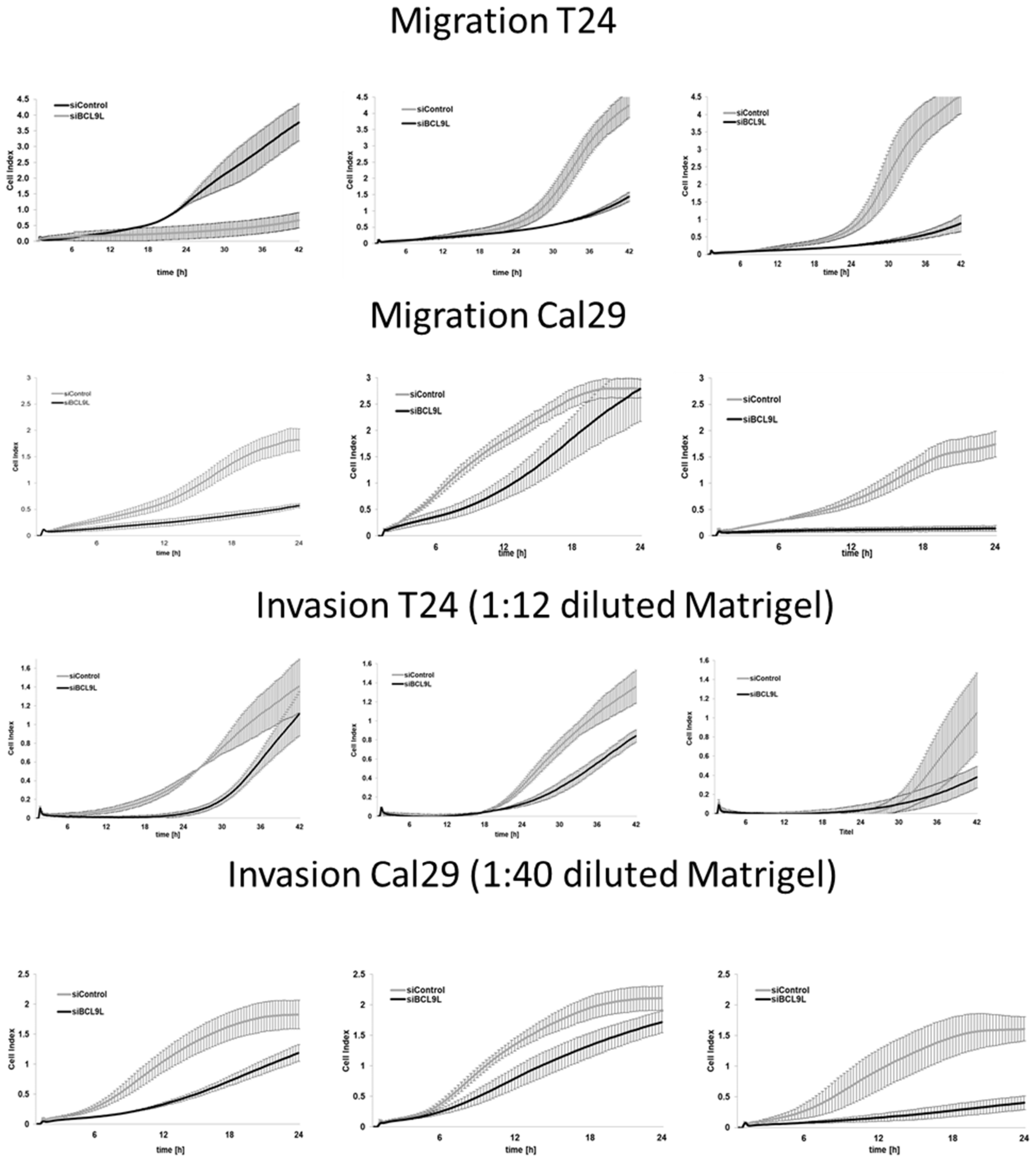

Supplementary Figure S4. *BCL9L*-dependent migration and invasion of Cal29 and T24 cells. Independent biological replicates of xCelligence migration and invasion assay. Migration of Cal29 and T24 is reduced after siRNA knockdown of *BCL9L*. Invasion was analyzed using 1:12 diluted matrigel for T24 and 1:40 diluted matrigel for Cal29. The invasiveness of T24 and Cal29 was significantly reduced after knockdown of *BCL9L*. The cell index values correspond to the cell number that migrates and invades. The data are expressed as mean ± standard deviation of two technical replicates.

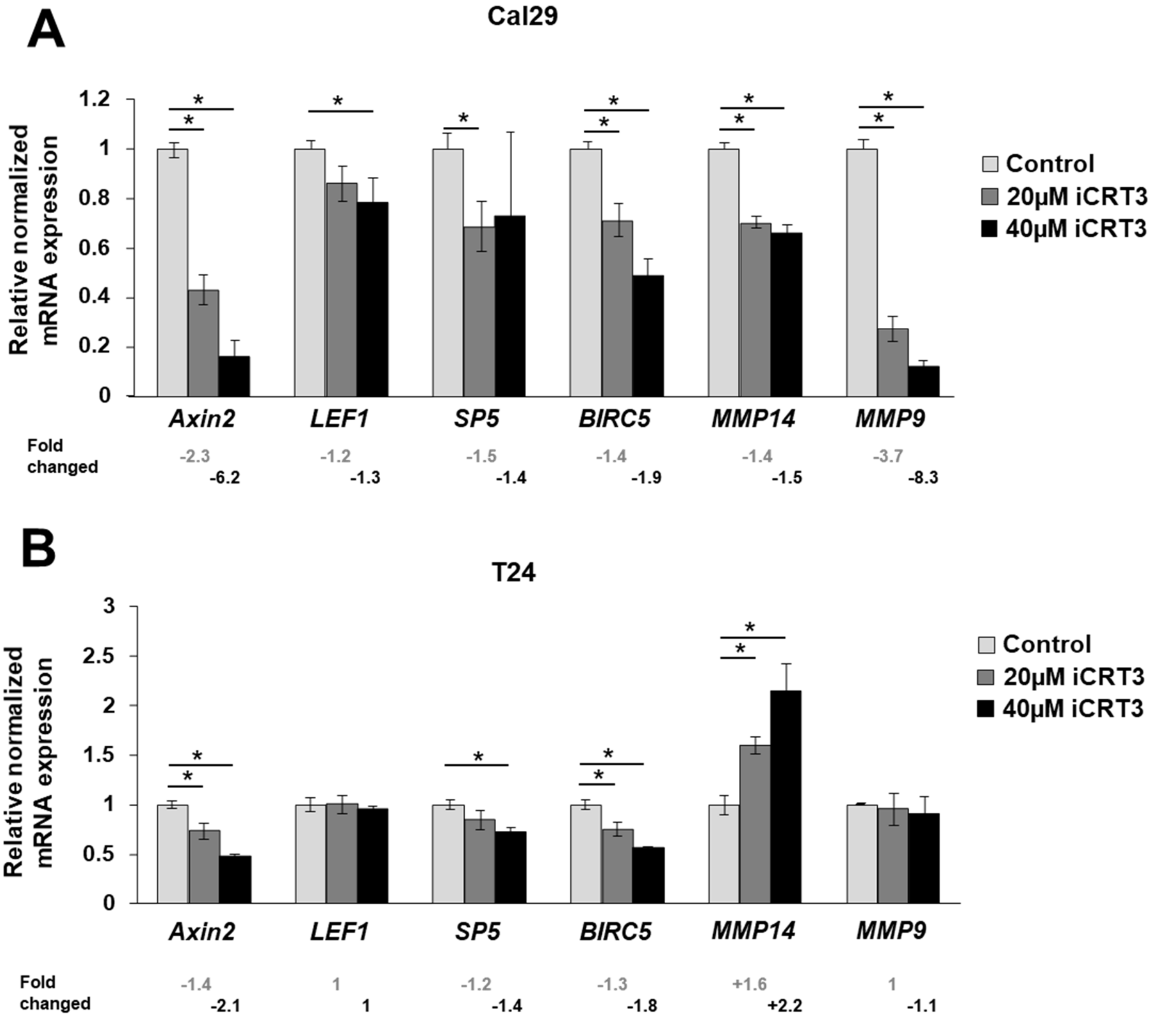

Supplementary Figure S5. Wnt/β-catenin target genes analysis by qRT-PCR after iCRT3 treatment. Cal29 (n=4) (A) and T24 (n=4) (B) cells were treated with 40 μM and 20 μM iCRT3, including 0.1 % DMSO control and 2 days later the mRNA expression of Wnt/β-catenin target genes *AXIN2*, *LEF1*, *SP5*, *BIRC5*, *MMP9* and *MMP14* were analysed by qRT-PCR. The mRNA expression is normalized to housekeeping genes *RPS13* and *RPS23* and are relative to DMSO control. Fold change isshown compared to DMSO control. The data are expressed as mean ± standard deviation and statistical analysis was performed by non-parametric Mann-Whitney U-test with * p-value < 0.05. n: independent biological replicates.

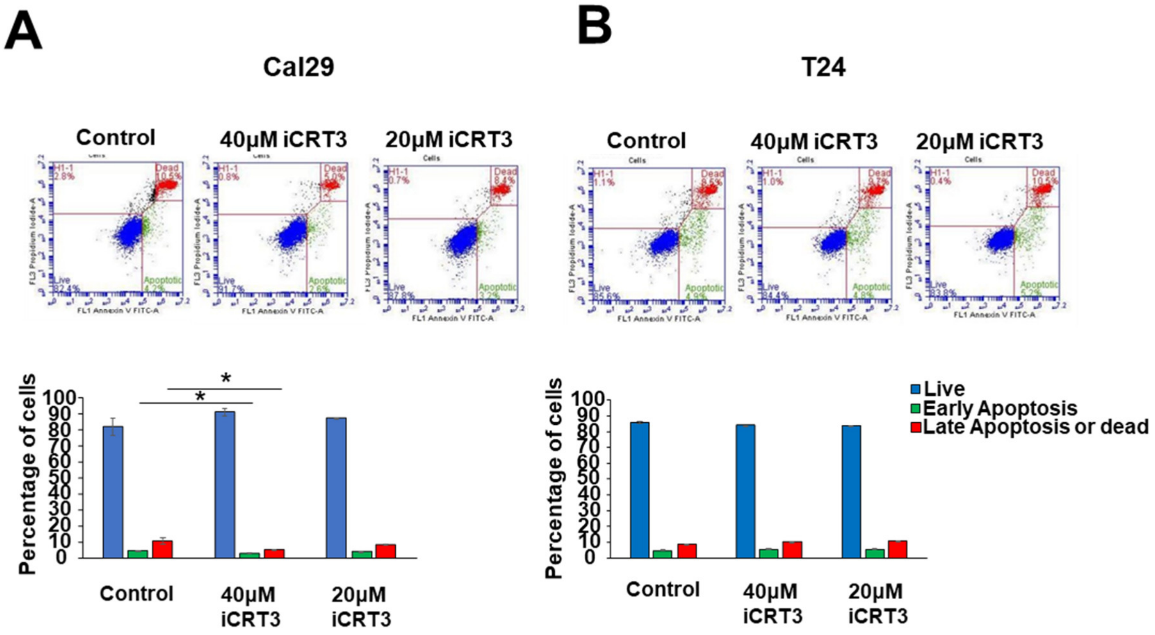

Supplementary Figure S6. Apoptosis analysis after Wnt/β-catenin signalling inhibition in bladder cancer cells. Cal29 (A) and T24 (B) cells were treated with 40 µM and 20 µM inhibitor iCRT3, including 0.1 % DMSO and after 2 days were analysed for apoptosis. 40 µM iCRT3 treatment slightly, however, significantly reduces early apoptosis and cell death or late apoptosis of Cal29 cells. No significant influence was observed between DMSO control and iCRT3 treatments for T24 cells. The apoptosis assay was performed by dual staining with annexin V-FITC and propidium iodide (PI) kit and analysed by flow cytometry. The data are expressed as mean ± standard deviation of three experiments and statistical analysis was performed by non-parametric Mann-Whitney U-test with * p-value < 0.05.

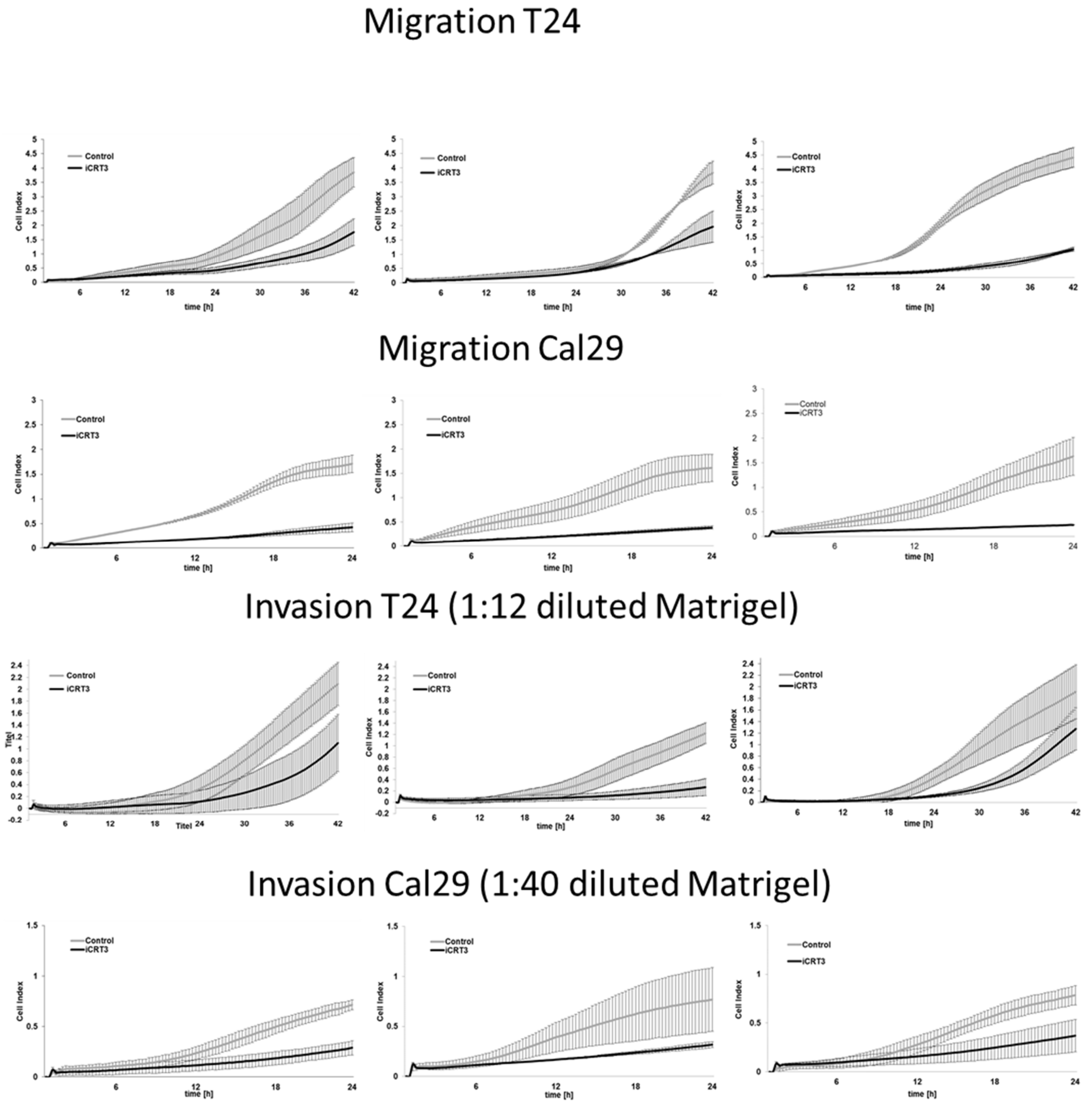

Supplementary Figure S7. iCRT3-dependent migration and invasion of Cal29 and T24 cells. Independent biological replicates of xCelligence migration and invasion assay. Migration of Cal29 and T24 is reduced after 40 µM iCRT3 treatment. Invasion was analyzed using 1:12 diluted matrigel for T24 and 1:40 diluted matrigel for Cal29. The invasiveness of T24 and Cal29 was significantly reduced after 40 µM iCRT3 treatment. The cell index values correspond to the cell number that migrates and invades. The data are expressed as mean ± standard deviation of three technical replicates.
